# Categorical versus continuous circulating tumor cell enumeration as early surrogate marker for therapy response and prognosis during docetaxel therapy in metastatic prostate cancer patients

**DOI:** 10.1186/s12885-015-1478-4

**Published:** 2015-06-09

**Authors:** Mark Thalgott, Brigitte Rack, Matthias Eiber, Michael Souvatzoglou, Matthias M. Heck, Caroline Kronester, Ulrich Andergassen, Victoria Kehl, Bernd J. Krause, Jurgen E. Gschwend, Margitta Retz, Roman Nawroth

**Affiliations:** 1 Department of Urology, Klinikum rechts der Isar, Technische Universität München, Ismaningerstraße 22, 81675 Munich, Germany; 2 Department of Gynecology and Obstetrics, Klinikum der Ludwig-Maximilians-Universität, Klinikum Innenstadt, Maistrasse 11, 80337 Munich, Germany; 3 Department of Radiology, Munich, Germany; 4 Department of Nuclear Medicine, Munich, Germany; 5 Institute of Medical Statistics and Epidemiology, Klinikum rechts der Isar, Technische Universität München, Ismaninger Str. 22, 81675 Munich, Germany; 6 Department of Nuclear Medicine, Universitätsklinikum Rostock, Schillingallee 35, 18057 Rostock, Germany

**Keywords:** Biomarkers, Circulating tumor cells, CTCs, Personalized treatment, Prostate cancer, Treatment efficacy

## Abstract

**Background:**

Circulating tumor cell (CTCs) counts might serve as early surrogate marker for treatment efficacy in metastatic castration-resistant prostate cancer (mCRPC) patients. We prospectively assessed categorical and continuous CTC-counts for their utility in early prediction of radiographic response, progression-free (PFS) and overall survival (OS) in mCRPC patients treated with docetaxel.

**Methods:**

CTC-counts were assessed in 122 serial samples, as continuous or categorical (<5 vs. ≥5 CTCs) variables, at baseline (q0) and after 1 (q1), 4 (q4) and 10 (q10) cycles of docetaxel (3-weekly, 75 mg/m2) in 33 mCRPC patients. Treatment response (TR) was defined as non-progressive (non-PD) and progressive disease (PD), by morphologic RECIST or clinical criteria at q4 and q10. Binary logistic and Cox proportional hazards regression analyses were used as statistical methods.

**Results:**

Categorical CTC-count status predicted PD at q4 already after one cycle (q1) and after 4 cycles (q4) of chemotherapy with an odds ratio (OR) of 14.9 (*p* = 0.02) and 18.0 (*p* = 0.01). Continuous CTC-values predicted PD only at q4 (OR 1.04, *p* = 0.048). Regarding PFS, categorical CTC-counts at q1 were independent prognostic markers with a hazard ratio (HR) of 3.85 (95 % CI 1.1-13.8, *p* = 0.04) whereas early continuous CTC-values at q1 failed significance (HR 1.02, 95 % CI 0.99-1.05, *p* = 0.14). For OS early categorical and continuous CTC-counts were independent prognostic markers at q1 with a HR of 3.0 (95 % CI 1.6-15.7, *p* = 0.007) and 1.02 (95 % CI 1.0-1.040, *p* = 0.04).

**Conclusions:**

Categorical CTC-count status is an early independent predictor for TR, PFS and OS only 3 weeks following treatment initiation with docetaxel whereas continuous CTC-counts were an inconsistent surrogate marker in mCRPC patients. For clinical practice, categorical CTC-counts may provide complementary information towards individualized treatment strategies with early prediction of treatment efficacy and optimized sequential treatment.

**Electronic supplementary material:**

The online version of this article (doi:10.1186/s12885-015-1478-4) contains supplementary material, which is available to authorized users.

## Background

In patients with metastatic castration resistant prostate cancer (mCRPC) first-line cytotoxic therapy with docetaxel is standard of care. About 45 % of patients are primary non-responders and tumor progression occurs after a median of 6–8 months [1–4]. Thus, early prediction of treatment efficacy is relevant for optimized and individualized treatment strategies, especially since recently newer agents like abiraterone, enzalutamide and radium-223 were established which may enable sequential treatment strategies and are applicable either prior or secondary to docetaxel [5–7].

During therapy, objective treatment response (TR) is routinely monitored with computed tomography (CT) and by additional bone scintigraphy with T-99 m labeled diphosphonates every four to six cycles of chemotherapy. Thus, the first response evaluation is performed 3–4 months after therapy initiation [6, 8]. Also, the prostate-specific antigen (PSA) value as a tumor marker requires a treatment interval of about 3 months to reach prognostic significance and is unreliable to reveal treatment response, as is the case with other serum derived markers such as lactate dehydrogenase and alkaline phosphatase [5, 9–12].

Consequently, there is a demand for a reliable predictive surrogate marker as an early indicator of treatment efficacy. A potential blood derived biomarker is the quantitative detection of circulating tumor cells (CTCs) that are highly investigated [13–18]. A US federal Food and Drug Administration (FDA) approved device for CTC-quantification and treatment monitoring in metastatic PC is the CellSearch™-System. Using this device, a threshold of ≥5 CTCs per 7.5 ml blood demonstrated prognostic significance for overall survival (OS) in metastatic PC [9–11].

There is evidence that CTC-counts are an early predictive surrogate marker for objective TR in breast and colorectal cancer [19–22]. Similarly in mCRPC patients elevated pre-treatment CTC-counts are associated with reduced radiologic response rates [23]. Therefore, we performed a prospective study in mCRPC patients, assessing longitudinal categorical and continuous CTC-count status during therapy in association with response assessments by radiographic RECIST- (Response Evaluation Criteria In Solid Tumors) and clinical criteria. The main objective was to compare the predictive and prognostic value of early categorical and continuous CTC-count status, after one cycle of chemotherapy for objective therapy response (TR), progression free (PFS) and overall survival (OS).

## Methods

**Fig. 1:**
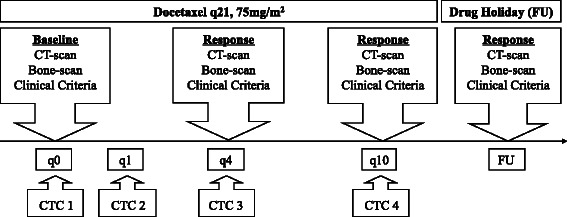
Study design

This prospective clinical validation study was approved by the ethics committee of the Klinikum rechts der Isar, Technische Universität München, Germany and was performed according to the ethical standards of the Declaration of Helsinki. All patients gave written informed consent. In total, 33 consecutive mCRPC patients undergoing first line docetaxel therapy (3-weekly, 75 mg/m2) were accrued from June 2008 to July 2010 at the Department of Urology, Technische Universität München, Germany. The main exclusion criterion was a second malignancy. Response monitoring was conducted by radiographic RECIST (rTR) and clinical criteria (cTR) after the fourth (q4) and tenth cycle (q10) of docetaxel and finally after two months of drug holiday (FU). CTCs were collected at baseline (q0) and after the first (q1), fourth (q4) and tenth (q10) cycle (Fig. 1). The main objective was to assess the predictive and prognostic value of early continuous and categorical CTC-counts for treatment response as well as progression free (PFS) and overall survival (OS).

### Blood analysis

Enumeration of CTCs was performed as described earlier using the CellSearch™-System (Veridex, Raritan, NJ, USA) according to the manufacturer’s protocol [9–11, 24, 25]. In brief, 7.5 ml blood was collected into CellSave™Preservative Tubes and processed within 96 h. Cells were isolated using the automated CellTracks®AutoPrep-System and CellSearch™Epithelial Cell Reagent Kit and identified with the CellSpotter™Analyzer. CTCs were characterized by EpCAM (epithelial cancer adhesion molecule) and staining for cytokeratin and nucleic acid as epithelial markers. CTCs were verified in a blinded fashion independently by two trained operators at the University of Munich, Germany. Routine laboratory analyses were performed at the clinical site, including PSA, lactate dehydrogenase, alkaline phosphatase and hemoglobin.

### Response evaluation

Response assessments were performed independently, with assessors blinded to CTC-results. A board certified radiologist evaluated the CT-scans according to RECIST 1.1 criteria for rTR, dichotomized into progressive disease (PD) or non-progressive disease (non-PD) [26]. Singular CT-scans were performed without intravenous contrast agent, e.g. due to renal insufficiency. In these cases RECIST criteria were used when the selected target lesions were measurable. If not, PD was defined by appearance of new metastases. For cTR, a genitourinary oncology physician subsumed the results of CT-scans and concomitant bone scans. Progression on bone scans was indicated by ≥2 new lesions [26, 27]. Additionally medical assessments including symptomatic progression and performance status as well as routine laboratory results like AP, LDH and PSA-values were taken into account with increasing levels indicating progression, similarly to Khoury et al. [11, 28, 29]. Based on cTR assessments treatment was aborted preterm in case of PD.

### Statistical Analysis

Sample sizes were calculated with a precision of ±20 % for an exact two-sided 95 % confidence interval for a proportion of 25 patients with detectable CTCs at baseline, amenable for response evaluation. The longitudinal course of CTC-counts as a continuous variable was analyzed as a function of the TR (PD vs. non-PD). Continuous CTC-counts were defined as absolute CTC-values in patients with PD and non-PD. Differences among groups and changes over time were compared using median CTC-count values. Differences were compared using the Mann–Whitney-Test and the predictive value for PD was assessed by binary logistic regressions. Secondary CTC-counts were handled as a categorical variable. Categorical CTC-counts were dichotomized according to the established threshold of <5 vs. ≥5 CTCs analogous to prognostic favorable and unfavorable CTC-counts described in the current literature and according to the FDA approved procedure [10]. Using this threshold, binary logistic regressions determined the predictive power for PD. Estimates of PFS and OS were analyzed using the Kaplan-Meier method, and differences thereof with the log-rank test. Cox proportional hazards regression analyses were used to determine the prognostic value of CTC-counts for PFS and OS. PFS was defined as the interval between baseline CTC-draw and the finding of a PD by rTR assessments or death and OS as the elapsed time from baseline CTC-collection to death or the last follow-up. PFS-time was limited by the defined FU-assessment. Tests were two-sided and were analyzed exploratory with α = 5 % using SPSS version 21 (SPSS Inc., Chicago, IL, USA).

## Results

### Patient demographics and response evaluation

The clinical characteristics of enrolled mCRPC patients are summarized in Table 1. Patients received a total of 235 cycles docetaxel with a median of 10 (3–10). Landmark analyses for CTC-counts were performed in a total of 122 samples. Concomitant objective rTR and cTR assessments were realized in 33 patients at q4, in 23 at q10 and 18 patients at FU. CTCs were detected in 27/33 (81.8 %, 95 % CI (64.5, 93.0)) of patients at baseline, in 24/33 (72.7 %, 95 % CI (54.5, 86.7)) at q1, in 20/33 (60.6 %, 95 % CI (42.1, 77.1)) at q4 and finally 14/23 (60.1 %, 95 % CI (38.5, 80.3)) at q10. Median intervals from CTC-collection at q0, q1 and q4 to rTR at q4 were 2.9 (2.4-4.7), 2.1 (1.7-3.66) and 0.0 months (0.0-0.4) while the interval from baseline to q10 or end of treatment was 7.2 months (4.0-9.0) and to FU 8.9 months (5.0-10.9). At the time of data analysis, 72.7 % (n = 24) of patients had died, resulting in a median OS of 10.7 months (95 % CI 5.8-15.6) and median PFS was 8.5 months (95 % CI 6.6-10.5).

**Table 1:** Clinical and pathological characteristics of the study cohort

Patients, n = 33	Value
Age (years)	
Median	70
Range	53-82
ECOG performance status, n (%)	
0	23 (69.6)
1	5 (15.2)
2	5 (15.2)
Gleason score at diagnosis, n (%)	
Unknown	3 (9.1)
≤ 7	11 (33.3)
≥ 8	19 (57.6)
Primary therapy, n (%)	
RP	9 (27.3)
EBRT	4 (12.1)
Palliative	20 (60.6)
Metastatic sites, n (%)	
Soft tissue	3 (9.0)
Bone + LN	15 (45.5)
Bone + Visceral ± LN	15 (45.5)
Biochemical markers^a^	
PSA [ng/dl]	140
	0.1-3378
Lactate dehydrogenase [U/l]	320
	218-1900
Alkaline phosphatase [U/l]	164
	50-1466
Hemoglobin [g/dl]	11.6
	8.5-16.1

*ECOG*, eastern cooperative oncology group; *EBRT*, external beam radiotherapy; *LN*, lymph node metastases; *PSA*, prostate-specific antigen; *RP*, radical prostatectomy; *n*, number

^a^values given as median and range

### Predictive power of categorical and continuous CTC-counts for therapy response

We further investigated the predictive power of early categorical CTC-counts (<5 vs. ≥5 CTCs) in logistic regression analyses (Table 2). Baseline CTC-values (q0) did not display predictive significance. In contrast, early unfavorable post-treatment CTC-counts at q1 were significantly predictive for PD at q4 with an odds ratio of 14.9 (p = 0.02) for rTR and 19.4 (p = 0.01) for cTR. In concomitant CTC-analyses at q4 and q10, the threshold of ≥5 CTCs was predictive for rTR, whereas for cTR only CTC-counts at q4 provided a predictive value (Table 2). Thus, early, categorical post-treatment CTC-counts <5 vs. ≥5 CTCs presented a powerful early indicator of treatment response with a median of 2.1 (1.7-3.7) months before the first routine response evaluation.

**Table 2:** Predictive power of continuous and categorical CTC-counts for therapy response

			Radiographic evaluation			Clinical criteria		
CTC-assessment	Interval	Patients (n=)	Odds Ratio	95 % CI	p=	Odds Ratio	95 % CI	p=
Continuous CTC-values	CTC q0 with OR at q4	33	1.0	0.99-1.02	0.53	1.0	0.99-1.04	0.17
	CTC q1 with OR at q4	33	1.03	0.99-1.07	0.17	1.1	0.99-1.13	0.06
	CTC q4 with OR at q4	33	1.04	1.0-1.09	0.048	1.1	1.03-1.26	0.009
	CTC q10 with OR at q10	23	1.02	0.98-1.07	0.28	1.1	1.0-1.23	0.052
Categorical CTC-counts	CTC q0 with OR at q4	33	6.5	0.7-60.5	0.1	8.0	0.86-74.2	0.07
	CTC q1 with OR at q4	33	14.9	1.6-142.2	0.02	19.4	2.0-185.7	0.01
	CTC q4 with OR at q4	33	18.0	1.9-174.2	0.01	24.0	2.5-233.5	0.006
	CTC q10 with OR at q10	23	16.3	1.4-183.1	0.02	5.0	0.82-30.5	0.08

q, cycle of docetaxel; *OR*, objective response

*p* values indicate statistical significance

Comparing early median values of continuous CTC-counts from patients with non-PD vs. PD at q4, as the earliest time point of TR evaluation, significant higher median CTC-counts were found in patients with PD at q0, q1 and q4 (Table 3). Focusing on the course of early continuous CTC-counts, we individually assessed CTC-kinetics in patients with PD or non-PD from q0 to q1 and q4. In both non-PD and PD patients a median CTC-decrease was found from q0 to q1. However, during the further course from q1 to q4, the CTC kinetic remained stable in patients with non-PD, whereas patients with PD displayed an increase in median CTC-counts (Table 3). Consequently early continuous CTC count status at q0 and q1 revealed no predictive value for PD at q4 in logistic regression analyses. In contrast, CTC-counts at q4 were significantly associated with PD assessed by concomitant rTR (*p* = 0.048) and cTR (*p* = 0.009). Interestingly, CTC-counts at q10 displayed no predictive significance (Table 2). Thus the continuous CTC-count status predicts treatment efficacy only when a therapy interval of about 12 weeks is reached.

**Table 3:** Variance of continuous CTC-counts according to therapy response after four (q4) or ten cycles (q10) of docetaxel

Response	CTC-Detection	Radiographic evaluation				Clinical criteria			
		q0^a^	q1^a^	q4	q10	q0^a^	q1^a^	q4	q10
non-PD	Median, Range	5	1	0	0	4.5	1	0	0
		0-225	0-85	0-115	0-73	0-97	0-38	0-29	0-71
	Patients, n=	25	25	25	17	24	24	24	14
PD	Median, Range	24	9.5	32	23	22	11	34	17
		4-97	1-58	3-358	0-36	4-225	1-85	3-358	0-73
	Patients, n=	8	8	8	6	9	9	9	9
Significance, p=		0.04	0.01	<0.001	0.03	0.03	0.004	<0.001	0.03

*PD*, progressive disease according to therapy response assessment at q4 or q10; q, cycle of docetaxel

^a^CTC-variance was assessed as a function of objective response at q4

### Prognostic value of categorical and continuous CTC-counts for progression free survival

According to the main objective of the study we focused on the prognostic value of early categorical CTC-counts (<5 vs. ≥5 CTCs) and early continuous CTC-counts in correlation with PFS and OS. PFS was analyzed according to morphologic RECIST criteria due to almost equal results for rTR and cTR in the association analyses.

**Fig. 2:**
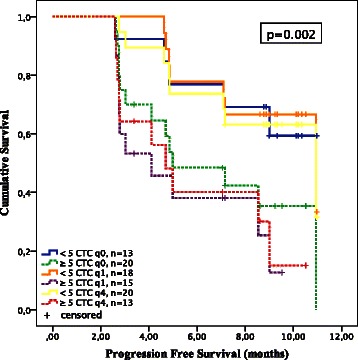
Kaplan Meier analyses for progression free survival (PFS) according to categorical CTC-counts (<5 vs. ≥5) at baseline (q0) and after one (q1) and 4 (q4) cycles of docetaxel

According to categorical CTC-counts, Kaplan Meier analyses revealed significant differences for PFS at each time point after treatment initiation when comparing patients with favorable (<5 CTCs) vs. unfavorable CTC-counts (≥5 CTCs) with a median of 10.9 (95 % CI 5.6-16.3) vs. 4.1 (95 % CI 1.5-6.7) months at q1 (p = 0.002) and 10.9 (95 % CI 5.5-16.3) vs. 4.7 (95 % CI 3.2-6.2) months at q4 (p = 0.01), respectively. Baseline CTC-counts were not prognostic for PFS (p = 0.09) (median PFS for patients with <5 CTCs not reached vs. 5.0 months (95 % CI 1.7-8.3) in patients with ≥5 CTCs) (Fig. 2). Consequently, for categorical post-treatment CTC-results at q1 and q4 the Cox proportional hazard ratio for PD was individually highly prognostic for PFS with 4.3 (95 % CI 1.6-11.8) and 3.2 (95 % CI 1.2-8.6). Similarly early continuous post-treatment CTC-counts at q1 and q4 revealed individual prognostic significance for PFS, but with a distinct lower hazard ratio of 1.02 (95 % CI 1.0-1.05) and 1.01 (95%CI 1.0-1.02) when compared to the values obtained for categorical CTC-counts. Baseline CTC-status was not predictive for PFS, neither by categorical nor by continuous CTC-assessments (Table 4).

**Table 4:** Cox proportional hazards regression analyses for progression free (PFS) and overall survival (OS) as a function of continuous and categorical (<5 CTCs vs. ≥5 CTCs) CTC-counts

			PFS			OS		
CTC-assessment	Interval	Patients (n=)	Hazard ratio	95 % CI	p=	Hazard ratio	95 % CI	p=
Continuous CTC-values	q0	33	1.01	0.99-1.02	0.4	1.01	0.999-1.01	0.07
	q1	33	1.02	1.0-1.05	0.04	1.02	1.01-1.04	0.01
	q4	33	1.01	1.0-1.02	0.004	1.02	1.01-1.03	0.001
	q10	23	1.02	0.99-1.04	0.3	1.03	1.01-1.05	0.004
Categorical CTC-counts	q0	33	2.4	0.9-6.8	0.1	3.8	1.4-10.3	0.009
	q1	33	4.3	1.6-11.8	0.005	4.5	1.9-10.8	0.001
	q4	33	3.2	1.2-8.6	0.02	5.8	2.2-15.1	<0.001
	q10	23	6.7	1.3-33.7	0.02	5.6	1.7-18.4	0.004

*p* values indicate statistical significance

### Prognostic value of categorical and continuous CTC-counts for overall survival

**Fig. 3:**
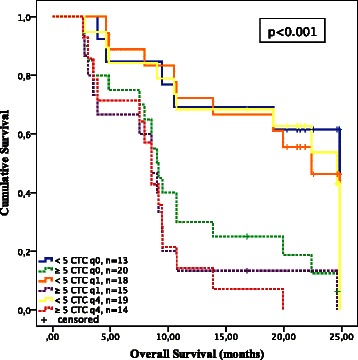
Kaplan Meier analyses for overall survival (OS) according to categorical CTC-counts (<5 vs. ≥5) at baseline (q0) and after one (q1) and 4 (q4) cycles of docetaxel

Regarding OS, categorical CTC-counts (<5 vs. ≥5 CTCs) significantly distinguished between favorable und unfavorable OS at each time point of CTC-sampling including baseline with a median OS of 24.8 (95% CI not defined) vs. 9.0 (95 % CI 7.7-10.4) months (q0; p = 0.005), and post-treatment values with a median OS of 22.4 (95 % CI 20.2-24.6) vs. 8.5 (95 % CI 6.6-10.5) months at q1 (p = 0.001) and a median OS of 24.6 (95 % CI 16.9-32.3) vs. 8.5 (95 % CI 7.5-9.6) months at q4 (p = 0.001) (Fig. 3). Consequently, Cox proportional hazard regression analyses revealed categorical CTC-counts highly prognostic for OS with a HR of 3.8 (95 % CI 1.4-10.3) at baseline and a HR of 4.5 (95 % CI 1.9-10.8) at q1 and 5.8 (95 % CI 2.2-15.1) at q4.

In contrast continuous baseline CTC-values failed significance (HR 1.01, 95 % CI 0.99-1.01, p = 0.07) whereas early continuous post-treatment CTC-counts at q1 and q4 revealed prognostic significance for OS with a low HR of 1.02 (95 % CI 1.01-1.04) and 1.02 (95 % CI 1.01-1.03), respectively (Table 4).

### Multivariate regression analyses

On multivariate Cox proportional hazards regression analyses analysis we compared categorical and continuous CTC-counts at the earliest time point of CTC-sampling after treatment initiation (q1) with concomitant routine laboratory analyses and a PSA-decline of ≥30 % (Table 5). Categorical CTC-counts at q1 were confirmed as independent predictors for PFS with a HR of 3.9 (95 % CI 1.1-13.8, p = 0.04). In contrast continuous CTC-values displayed no independent prognostic value for PFS with a HR of 1.02 (95 % CI 0.99-1.05, *p* = 0.14). Concerning cox regression analysis for OS, both categorical CTC-counts and continuous CTC-counts revealed an independent significant prognostic value for OS with a HR of 4.9 (95 % CI 1.6-15.7, *p* = 0.007) and 1.02 (95 % CI 1.0-1.04, p = 0.04), respectively.

**Table 5:** Multivariate Cox proportional hazards regression for progression free (PFS) and overall survival (OS) in a model including post-treatment laboratory analyses after one cycle of docetaxel (q1) with either continuous or categorical CTC-counts

		PFS			OS		
CTC-assessment	Values in model	Hazard ratio	95 % CI	p=	Hazard ratio	95 % CI	p=
Continuous CTC-values	CTC-value Continuous	1.02	0.99-1.05	0.14	1.02	1.01-1.04	0.04
	PSA-value ≥30 % decline	1.8	0.6-5.6	0.3	2.6	0.9-7.78	0.09
	Lactate dehydrogenase ≤ ULN vs. >ULN	1.2	0.2-10.2	0.8	0.7	0.1-3.4	0.65
	Alkaline phosphatase ≤ ULN vs. >ULN	3.2	0.95-10.99	0.06	3.4	1.1-10.3	0.03
	Hemoglobin < LLN vs. ≥LLN	2.7	0.3-28.4	0.4	1.7	0.2-17.0	0.63
Categorical CTC-counts	CTC-count <5 CTCs vs. ≥5 CTCs	3.9	1.1-13.8	0.04	4.9	1.6-15.7	0.007
	PSA-value ≥30 % decline	1.8	0.6-5.3	0.3	3.0	1.03-8.8	0.04
	Lactate ehydrogenase ≤ ULN vs. >ULN	1.5	0.2-11.8	0.7	0.8	0.2-3.9	0.84
	Alkaline phosphatase ≤ ULN vs. >ULN	1.9	0.5-7.4	0.4	2.0	0.6-6.5	0.27
	Hemoglobin < LLN vs. ≥LLN	3.3	0.3-34.3	0.3	2.2	0.2-21.0	0.5

### Exploratory analyses for the prognostic value of CTC-dynamics

As presented in Additional files 1, 2, 3 and 4 we performed further exploratory analyses investigating early CTC-dynamics from q0 to q1. We could demonstrate the conversion of CTC-counts below the established threshold of 5 CTCs relevant for PFS and OS. Similarly, a 50 % decrease algorithm as a potential measure of continuous CTC-value changes revealed prognostic impact on OS and PFS. Assessing the 50 % decrease, 44 % of the patients reaching the 50 % decline in CTC-counts simultaneously demonstrated a CTC-count decrease below the threshold of 5 CTCs.

## Discussion

In this prospective clinical trial we examined the predictive and prognostic value of early CTC-count status for monitoring treatment efficacy in mCRPC patients. To the best of our knowledge this is the first study that comparatively investigates the predictive and prognostic value of early continuous vs. categorical CTC-count status for therapy response in a homogenous cohort of mCRPC patients, assessing several defined longitudinal time points during chemotherapy with docetaxel. According to our data, categorical CTC-count status relative to a threshold of 5 CTCs is an early predictor of treatment response, already at the end of the first cycle of chemotherapy, 9–12 weeks before the first radiologic objective response assessment. In contrast early continuous CTC-values displayed no early predictive value for therapy response by morphologic RECIST or clinical criteria. The predictive value of early categorical post-treatment CTC-count status for therapy response was similarly observed in metastatic breast and colorectal cancer [19–21, 30]. In our cohort pretreatment CTC-counts were not associated with therapy response. In contrast earlier results in mCRPC-patients revealed an association of elevated baseline CTC-counts (≥5 CTCs) with reduced radiologic and biochemical response while the post-treatment CTC-dynamic revealed an association with PSA-response [23].

Focusing on the predictive value of the continuous CTC-count status for therapy response, early continuous CTC-values at q1 were not predictive for objective response, although the comparison of median absolute CTC-counts significantly differed in patients with PD vs. non-PD at all time points. The missing predictive value of continuous CTC-counts might be reflected by the CTC-kinetics. After one cycle of chemotherapy early CTC-kinetics revealed a decrease of median CTC-values in non-PD and PD patients alike. According to rTR in non-PD patients the initial median CTC decrease was 80 % (p = 0.002) and in PD patients 60.4 % (p = 0.1) and for cTR 77.7 % (p = 0.002) and 50 % (p = 0.086). However, at later stages during therapy an increase of median CTC-counts was measured only in patients with PD. Therefore, continuous CTC-counts seem to require an interval of up to 12 weeks to reflect therapy response in imaging devices. To our knowledge we are the first to present analyses of continuous CTC-count status in relation to radiologic response, during a defined course of docetaxel in mCRPC patients. Earlier studies investigated the association and predictive value of directional or delta changes of log10 transformed CTCs with clinical response in overall interval-adjusted analyses [28, 29]. In CRPC-patients mean changes of CTC-counts were not predictive for clinical PD, neither by unidirectional nor by delta changes despite a trend for increasing CTC-counts in patients with PD and relatively unchanged values in non-PD. In this cohort mean changes in CTC-counts did not differ significantly between patients with PD vs. non-PD [28]. In contrast, investigating a mixed cohort of castration sensitive (CSPC) and mCRPC patients, an average CTC-decrease in non-PD and a CTC-increase in PD patients was observed, with a significant individual predictive value of CTC-value changes for the risk of clinical progression. Concordance analyses of directional CTC-changes with clinical outcome revealed a sensitivity of 79 % and a specificity of 75 % [29]. Similarly Goodman et al. demonstrated in analyses conducted on log10 transformation of CTC-counts that elevated CTC numbers during treatment were associated with a higher risk for transition from HSPC to CRPC. In this study only baseline CTC-counts were independently prognostic for the risk of CRPC [31].

Thus, there is an inconsistent body of evidence concerning whether continuous CTC-counts might have predictive value for therapy response. Our results indicate that continuous CTC-counts as a predictive marker require sampling at multiple time points over a period of up to 12 weeks. This is in accordance with a larger study that included different types of cancers, and which demonstrated that about 3 months are needed to monitor the treatment effect on continuously assessed CTCs [32]. Comparing different concepts of % change in CTCs with a defined % reduction confidence or fold changes with absolute and proportional reduction cutoffs vs. categorical CTC-enumeration demonstrated that a static CTC cutoff is the best method to determine whether a therapy is effective [32]. Similarly a fold change in CTC-counts was only moderately associated with survival time and proportional changes of continuous CTC-counts with ≥30 % or ≥50 % revealed conflictive results with respect to survival [11, 23, 27].

Focusing on PFS we could demonstrate that early categorical posttreatment CTC-counts were highly prognostic, as was demonstrated earlier for other carcinomas [19, 20, 30, 33]. In addition, we demonstrated categorical CTC-counts, after the first cycle of docetaxel, as an independent prognostic marker for PFS. Interestingly, in our study baseline CTC-values did not display prognostic value for PFS. This contrasts earlier results in CSPC patients, where unfavorable categorical baseline and posttreatment CTC-counts were associated with reduced time to castration-refractory stages, but is in accordance with results in metastatic colorectal cancer [20, 31, 34]. Investigating the continuous CTC-count status as a prognostic tool for PFS, in our study post-treatment CTC-values displayed a significant prognostic value, despite a distinct lower hazard ratio when compared to categorical CTC-counts. Consequently early continuous CTC-counts after one cycle of docetaxel were not independently prognostic for PFS. Therefore early categorical posttreatment CTC-counts seem to be a superior prognostic marker for PFS, when compared to the continuous CTC-value status.

In our study response was primary defined by the objective radiologic response (rTR) performed by RECIST 1.1 criteria and secondary by clinical response (cTR). Both modalities were rated blinded to CTC-assessments. With regard to the selected RECIST 1.1 criteria, we handled bone metastases as non-measurable lesions as all of the bone metastases being present in the patients examined by our study show no measurable soft-tissue component. Patients with non-measurable disease only at baseline were allowed, with a defined PD in case of an increased tumor burden or a substantial worsening as defined by RECIST 1.1 [26]. This approach is in accordance with earlier studies using RECIST criteria but might be limited due to a potential misinterpretation of a radiographic flare response with sclerosing of osseous metastases as PD. It is well known that under various therapies in bone metastases an increased activity of osteoblasts can occur resulting in an higher density in CT which could be misinterpreted as progressive disease [11, 31, 35]. However, it has to be taken into account that the interpretation as PD defined by RECIST 1.1 requires an uniequivocal progression with a total increase in tumor burden. An only modest increase is not regarded as enough for PD in non-target lesions. This approach as conducted in our study limits, however not completely omits the incorrect classification of patients with stable disease as PD. In our trial the additionally evaluated clinical response (cTR) revealed similar results when compared to rTR. Similar to earlier studies cTR was assessed mainly based on imaging findings with RECIST evaluations of CT-scans and concomitant bone scans where new lesions (≥2) indicated progression [26–28]. Supplementary, medical assessments including symptomatic progression and performance status were taken into account, and to a lesser extent laboratory studies with increasing PSA, LDH and AP levels indicating progression [11, 28]. Beside the objective rTR evaluation by RECIST criteria, the cTR assessment in our study may incorporate a subjective bias and represents a limitation. Therefore further studies should apply defined weight bearing percentages for the individual clinical criteria as demonstrated by Gonzales et al. or should consider the recommendations of the prostate cancer clinical trials working group (PCWG2 guidelines) for response assessment [26, 27, 29].

Focusing on OS, categorical CTC-count status displayed a significant association with OS for each time point during therapy including baseline. Early categorical CTC-counts at q1 were confirmed as an independent prognostic surrogate for OS. Thus, our data complement other studies in which the prognostic value of a defined CTC-threshold for OS has been demonstrated for baseline and 2–5 weeks after therapy induction or during later intervals [10, 11, 23, 34, 36]. Although the delineated threshold for survival varies across studies from 3 to 5 CTCs [9–11, 24, 31, 34, 36–38] in our study the FDA-approved threshold for therapy monitoring of <5 vs. ≥5 CTCs was applicable as a predictor of treatment efficacy in our cohort. Regarding continuous CTC-values our study revealed also the early posttreatment continuous CTC status as an independent prognostic marker for OS at q1 despite a lower hazard ratio when compared to categorical CTC-counts. These results supplement earlier data by Scher et al. demonstrating that elevated continuous CTC-values were associated with a higher risk of death and decreased survival [27].

As presented in additional files (Additional files 1, 2, 3 and 4) we performed further exploratory analyses investigating early CTC-dynamics from q0 to q1. We could demonstrate the conversion of CTC-counts below the established threshold of 5 CTCs relevant for PFS and OS, as was demonstrated earlier for OS [10, 11, 23]. Similarly, a 50 % decrease algorithm as a potential measure of continuous CTC-value changes revealed prognostic impact on OS and PFS. Assessing the 50 % decrease, 44 % of the patients reaching the 50 % decline in CTC-counts simultaneously demonstrated a CTC-count decrease below the threshold of 5 CTCs. Our results complement initial trials demonstrating that not only the conversion of CTC-counts to a favorable level of <5 CTCs, but also a percent decrease shows a prognostic value for survival in mCRPC patients as it was demonstrated initially for OS by using a proportional fall of ≥30 % of continuously assessed CTC-counts [11]. In contrast a recent study demonstrated no significant association of a 50 % decrease with OS [23]. Thus there is inconsistent evidence regarding the prognostic value of continuous CTC-values for survival whereas the prognostic value of categorical CTC-counts was confirmed in a broad range of trials with respect to OS. Taken together categorical CTC-counts seem to be a superior surrogate marker when compared to continuous CTC-values presenting in our study a higher hazard ratio for survival and a clinically easily applicable threshold with an early predictive value for objective treatment response. In addition categorical CTC-counts displayed an early independent value for PFS and OS, applicable already after one cycle of chemotherapy, up to 3.7 months before the first imaging staging procedures. Therefore, the aim of therapies should be the conversion of CTC-counts to favorable values, as has been suggested also by others [32]. Limiting our results might be attributed to the small patient cohort and need to be confirmed in a large cohort especially with regard to randomized CTC-guided treatment strategies. In addition further studies need to address the molecular characterization of CTCs as a liquid biopsy [39–42].

## Conclusions

Our data give evidence for early categorical CTC-count enumeration, but not for continuous CTC-values, as a predictive molecular surrogate marker for treatment response in mCRPC patients up to 3.7 months before the first radiographic evaluation. In addition our findings suggest early categorical posttreatment CTC-count status (<5 vs. ≥5 CTCs), already after one cycle of therapy, as an independent prognostic marker for PFS and OS, whereas continuous CTC-values displayed an inconsistent prognostic value.

## Additional files

Additional file 1:
**Kaplan Meier analyses for progression free survival in dependency of early CTC-dynamics relative to the threshold of 5 CTCs (<5 vs. ≥5) for the interval from baseline (q0) to the end of the first cycle docetaxel (q1).**

Additional file 2:
**Kaplan Meier analyses for progression free survival in dependency of early CTC-dynamics relative to a CTC-count decrease of ≥50 % for the interval from baseline (q0) to the end of the first cycle docetaxel (q1).**

Additional file 3:
**Kaplan Meier analyses for overall survival (OS) according to CTC-dynamics relative to a threshold of 5 CTCs (<5 vs. ≥5) for the interval from baseline (q0) to the end of the first cycle docetaxel (q1).**

Additional file 4:
**Kaplan Meier analyses for overall survival (OS) according to CTC-dynamics relative to a CTC-count decrease of ≥50 % for the interval from baseline (q0) to the end of the first cycle docetaxel (q1).**

## Abbreviations

CI: Confidence interval
CSPC: Castration sensitive prostate cancer
CT: Computed tomography
CTCs: Circulating tumor cells
cTR: Clinical treatment response
EpCAM: Epithelial cancer adhesion molecule
FDA: US federal Food and Drug Administration
FU: Follow up
HR: Hazard ratio
mCRPC: Metastatic castration-resistant prostate cancer
non-PD: Non-progressive disease
OR: Odds ratio
OS: Overall survival
PC: Prostate cancer
PD: Progressive disease
PFS: Progression free survival
PSA: Prostate-specific antigen value
q: Cycle of docetaxel treatment
RECIST: Response evaluation criteria in solid tumors
rTR: Radiographic treatment response
TR: Treatment response
