# Supplementary material for: Categorical versus continuous circulating tumor cell enumeration as early surrogate marker for therapy response and prognosis during docetaxel therapy in metastatic prostate cancer patients
Source: BMC Cancer. 2015 Jun 9;15:458. doi: 10.1186/s12885-015-1478-4 (PMC4459665; doi:10.1186/s12885-015-1478-4)
Supplement: Additional file 1: — Kaplan Meier analyses for progression free survival in dependency of early CTC-dynamics relative to the threshold of 5 CTCs (<5 vs. ≥5) for the interval from baseline (q0) to the end of the first cycle docetaxel (q1). [file 12885_2015_1478_MOESM1_ESM.pdf]

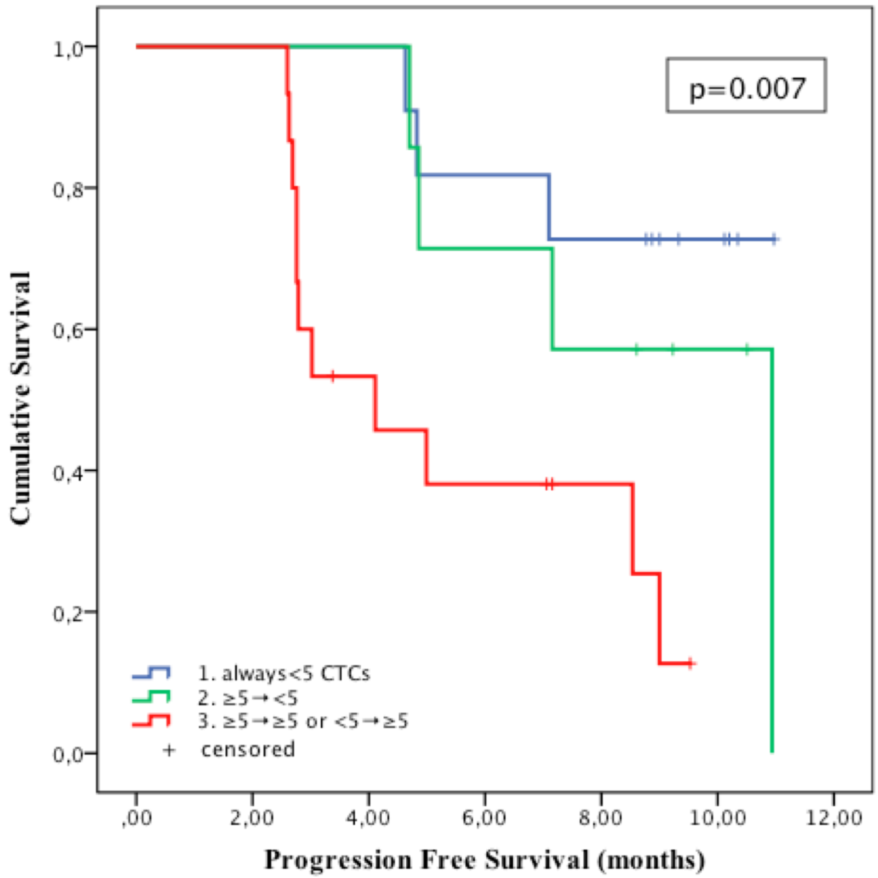

**Additional file 1:** Kaplan Meier analyses for progression free survival in dependency of early CTC-dynamics relative to the threshold of 5 CTCs (<5 vs. ≥5) for the interval from baseline (q0) to the end of the first cycle docetaxel (q1).					
Groups	CTC-counts	Patients, n=	PFS, months	95%CI	p=
Group 1	always <5	11	n.a.	n.a.	1 vs. 2: 0.4<br>1 vs. 3: 0.006<br>2 vs. 3: 0.07
Group 2	≥5 → <5	7	10.9	n.a.	
Group 3	≥5 → ≥5 or <5 → ≥5	15	4.1	1.5-6.7	
