# Supplementary material for: Categorical versus continuous circulating tumor cell enumeration as early surrogate marker for therapy response and prognosis during docetaxel therapy in metastatic prostate cancer patients
Source: BMC Cancer. 2015 Jun 9;15:458. doi: 10.1186/s12885-015-1478-4 (PMC4459665; doi:10.1186/s12885-015-1478-4)
Supplement: Additional file 2: — Kaplan Meier analyses for progression free survival in dependency of early CTC-dynamics relative to a CTC-count decrease of ≥50 % for the interval from baseline (q0) to the end of the first cycle docetaxel (q1). [file 12885_2015_1478_MOESM2_ESM.pdf]

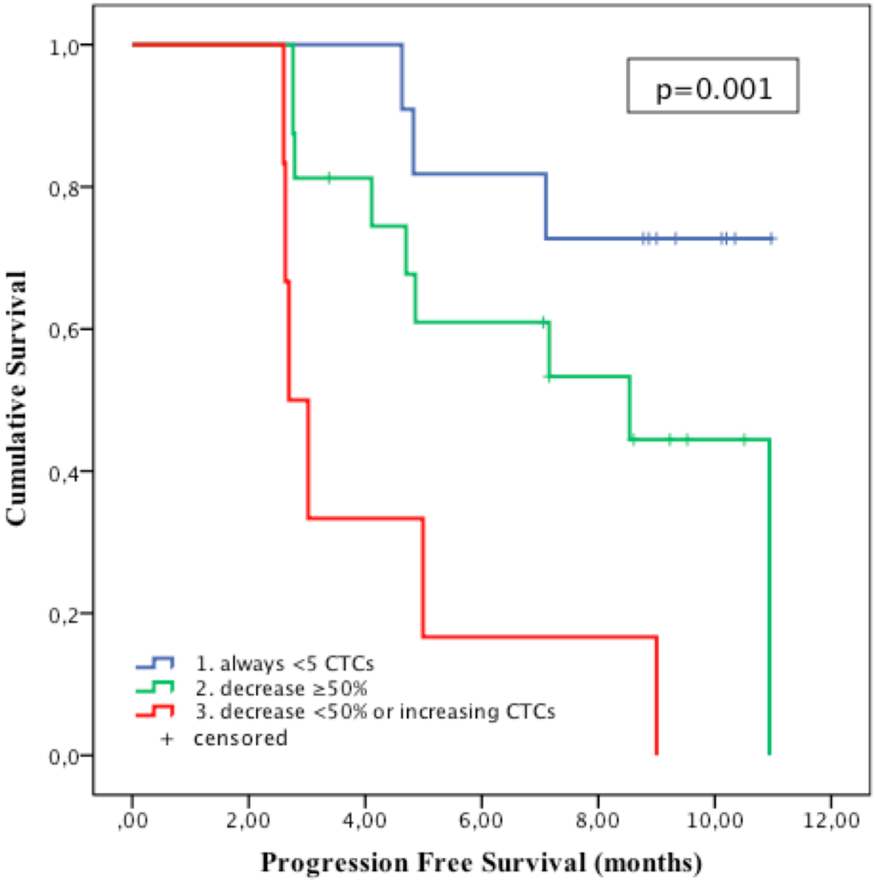

Additional file 2: Kaplan Meier analyses for progression free survival in dependency of early CTC-dynamics relative to a CTC-count decrease of ≥50% for the interval from baseline (q0) to the end of the first cycle docetaxel (q1).

Groups	CTC-counts	Patients, n	PFS, months	95%CI	p
Group 1	always <5	11	n.a.	n.a.	1 vs. 2: 0.1<br>1 vs. 3: 0.001<br>2 vs. 3: 0.01
Group 2	decrease ≥50%	16	8.5	2.6-14.5	
Group 3	decrease <50% or increasing	6	2.7	2.2-3.2	
