# Supplementary material for: Categorical versus continuous circulating tumor cell enumeration as early surrogate marker for therapy response and prognosis during docetaxel therapy in metastatic prostate cancer patients
Source: BMC Cancer. 2015 Jun 9;15:458. doi: 10.1186/s12885-015-1478-4 (PMC4459665; doi:10.1186/s12885-015-1478-4)
Supplement: Additional file 3: — Kaplan Meier analyses for overall survival (OS) according to CTC-dynamics relative to a threshold of 5 CTCs (<5 vs. ≥5) for the interval from baseline (q0) to the end of the first cycle docetaxel (q1). [file 12885_2015_1478_MOESM3_ESM.pdf]

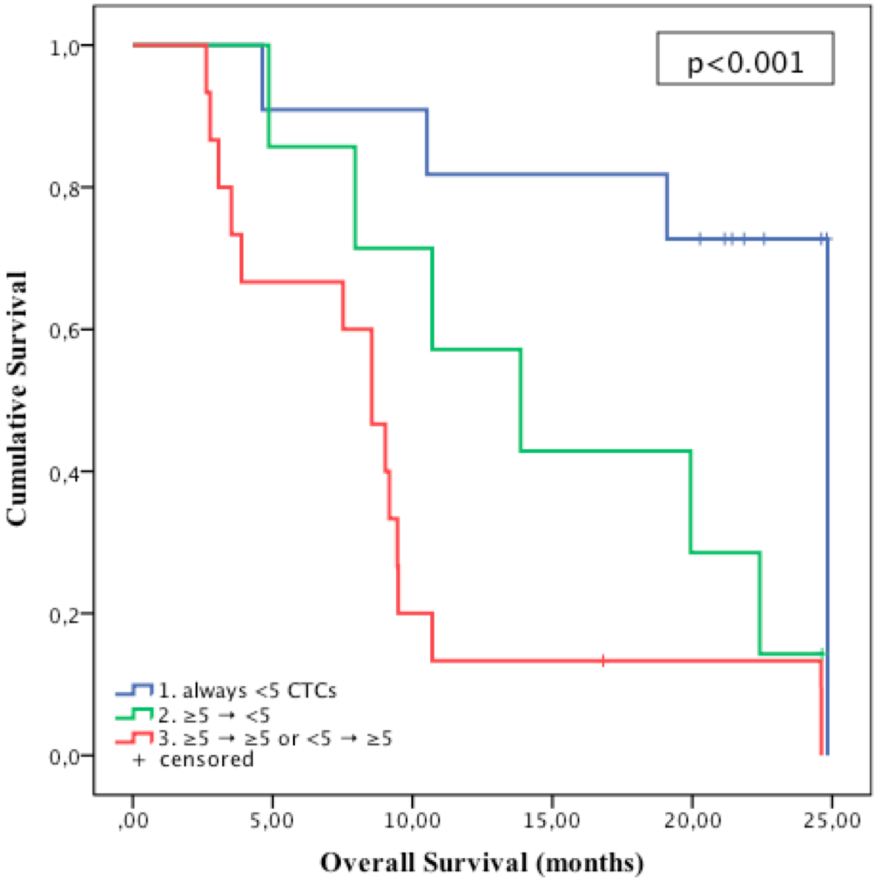

Additional file 3: Kaplan Meier analyses for overall survival (OS) according to CTC-dynamics relative to a threshold of 5 CTCs (<5 vs. ≥5) for the interval from baseline (q0) to the end of the first cycle docetaxel (q1).

Groups	CTC-counts	Patients, n	OS, months	95%CI	p
Group 1	always <5	11	24.8	n.a.	1 vs. 2: 0.03<br>1 vs. 3: <0.001<br>2 vs. 3: 0.11
Group 2	≥5→<5	7	13.9	5.8-21.9	
Group 3	≥5 → ≥5 or <5 → ≥5	15	8.5	6.6-10.5	
